# Liquid lncRNA Biopsy for the Evaluation of Locally Advanced and Metastatic Squamous Cell Carcinomas of the Head and Neck

**DOI:** 10.3390/jpm10030131

**Published:** 2020-09-16

**Authors:** Izabela Łasińska, Tomasz Kolenda, Kacper Guglas, Magda Kopczyńska, Joanna Sobocińska, Anna Teresiak, Norbert Oksza Strzelecki, Katarzyna Lamperska, Andrzej Mackiewicz, Jacek Mackiewicz

**Affiliations:** 1 Department of Medical and Experimental Oncology, Heliodor Swiecicki Clinical Hospital, Poznan University of Medical Sciences, 16/18 Grunwaldzka Street, 60-786 Poznan, Poland; 2 Specialist Nursing Laboratory, Faculty of Medicine and Health Science, University of Zielona Góra, Energetyków Street 2, 65-00 Zielona Gora, Poland; 3 Department of Cancer Immunology, Chair of Medical Biotechnology, Poznan University of Medical Sciences, 8 Rokietnicka Street, 60-806 Poznan, Poland; mg.kopczynska@gmail.com (M.K.); a.s.sobocinska@gmail.com (J.S.); norbert@strzelecki.org (N.O.S.); mackiewicz.aa@gmail.com (A.M.); 4 Laboratory of Cancer Genetics, Greater Poland Cancer Centre, 15 Garbary Street, room 5025, 61-866 Poznan, Poland; kacper.guglas@gmail.com (K.G.); anna.teresiak@wco.pl (A.T.); kasialam@o2.pl (K.L.); 5 Postgraduate School of Molecular Medicine, Medical University of Warsaw, 61 Zwirki i Wigury Street, 02-091 Warszawa, Poland; 6 Department of Diagnostics and Cancer Immunology, Greater Poland Cancer Centre, 15 Garbary Street, 61-866 Poznan, Poland; 7 Department of Oncology, Poznan University of Medical Sciences, 82-84 Szamarzewskiego, 60-569 Poznan, Poland

**Keywords:** head and neck cancer, HNSCC, lncRNA, liquid biopsy, personalized medicine, biomarker, metastasis, palliative chemotherapy

## Abstract

**Background:** Long non-coding RNA (lncRNA) are RNA molecules that are more than 200 nucleotides long and have the ability to modify the activity of genes. They can be found in both healthy and cancer tissues, as well as in plasma, saliva and other bodily fluids. They can also be used as biomarkers of early detection, prognosis and chemotherapy resistance in several cancer types. Treatment of head and neck squamous cell carcinoma (HNSCC) patients with locally advanced disease is still difficult, and choice of treatment should be based on more precise and available biomarkers, such as those obtained from a liquid biopsy. For improvement of treatment efficacy, identification and clinical implementation of new biomarkers are of the utmost importance. **Methods:** Plasma samples drawn before (p1) and three cycles post (p2) (TPF: docetaxel, cisplatin, 5-fluorouracil/PF: cisplatin, 5-fluorouracil) chemotherapy from 53 HNSCC patients (17 with locally advanced and 36 with metastatic disease) and 14 healthy volunteers were studied. Expression levels of 90 lncRNA expression were analyzed using the qRT-PCR method, and the obtained results were compared between proper groups. Statistical analyses were carried out using Jupyter Notebooks (5.7.2), Python (ver. 3.6) and GraphPad Prism 8. **Results:** The study demonstrated the differences between the expressions of several lncRNA in cancer patients’ and healthy volunteers’ plasma, as well as between locally advanced and metastatic patients’ groups. A correlation between the response to systemic therapy and lncRNA expression levels was observed. Patients with a (high/low) expression of Alpha 250 and Emx2os showed statistically significant differences in progression free survival (PFS), as well as for overall survival (OS) depending on the level of Alpha 250, snaR, SNHG1. The univariate and multivariate Cox regression model showed Alpha 250 as the best prognostic factor for HNSCC patients. **Conclusions:** Liquid biopsies based on lncRNAs are promising diagnostic tools that can be used to differentiate between those with cancer and healthy individuals. Additionally, they can also serve as biomarkers for chemotherapy resistance. An identified, circulating lncRNA Alpha 250 seems to prove the best prognostic biomarker, associated with extended PFS and OS, and should be validated in a larger cohort in the future.

## 1. Introduction

Head and neck cancers are mainly squamous cell carcinomas (HNSCCs, head and neck squamous cell carcinomas) with approximately 529,000 new cases diagnosed worldwide in 2012 [1]. The etiology of HNSCCs is complex and depends mostly on external risk factors, such as tobacco and/or alcohol use in older patient groups and infection with human papillomavirus (HPV) in younger patients. Furthermore, Epstein–Barr virus infection has been shown to play a role in the case of nasopharyngeal cancers [2]. According to The Cancer Genome Atlas (TCGA) the main genetic alterations present in HNSCC are associated with protein-coding genes involved in cell cycle (CDKN2A, TP53, CCND1), growth signaling (EGFR), survival (PIK3CA, PTEN), WNT signaling (FAT1, AJUBA, NOTCH1) and epigenetic regulation (KMT2D, NSD1). In human papillomavirus (HPV) infection, tumors showed changes in p16INK4A, TP53, RB or PI3CA [3].

Modern sequencing methods, as well as the creation and sharing of databases, enable faster and easier searching for molecular markers which can be used for HNSCC diagnostic, prognostic and prediction purposes [4]. The improvement of HNSCC treatment is still something to be achieved and current investigations are focusing on immunotherapy and specific biomarkers such as PD-L1/PD-L2 expression or the interferon-gamma gene signature [5]. However, a large part of the research on biomarkers in HNSCC concerns a transcriptome that does not encode a protein, which includes regulatory RNAs such as miRNA or lncRNA [6,7]. Moreover, when it comes to HNSCC serum, saliva and plasma are good sources of potential biomarkers [8].

It is accepted that only about 2% of the human genome encodes proteins, while the remaining 98% is classified as ‘junk’ DNA [9,10]. Further studies have shown that ‘junk’ DNA might be processed as RNA with non-protein coding potential (non-coding RNA, ncRNA) and play a role in cellular processes. It can also serve as a regulator of gene action as part of epigenetic mechanisms [11,12,13]. The changes in ncRNAs are the important elements of tumorigenesis and cancer progression [14]. One of the known types of ncRNAs is long non-coding RNAs (lncRNAs). lncRNAs are longer than 200 nucleotide transcripts. They can modify histone complexes and nucleus architecture and have an impact on RNA stability through epigenetic modifications, RNA transcription, translation and post-translational modification. Additionally, they interact with signaling and regulatory proteins and they act as “molecular sponges” [15,16]. There are about 50,000 types of lncRNAs in humans, which are transcribed mostly from introns. They are characterized by low conservatives across our species [17,18]. lncRNA transcripts are located in the nucleus, cytoplasm, or when circulating molecules form in various bodily fluids, such as plasma, saliva or gastric fluid [19,20,21]. It is estimated that about 40–90% of lncRNAs are translated, but these proteins are likely unstable [22].

Different lncRNAs take part in many important cellular processes, such as cell cycle, response to stress (including response to chemotherapeutics and irradiation), cellular senescence, cellular communication, hematopoiesis, embryogenesis, virus–host interactions and tissue microenvironment modification [23,24,25,26,27,28,29]. In cancerogenesis, changes in the expression levels of lncRNA are reported in tissues, as well as in bodily fluids [7]. lncRNA are responsible for the invasion, proliferation and metastatic potential in cancer cells as well as apoptosis in many cancers [15,30,31].

In HNSCC, more and more lncRNAs are linked with the biology of this cancer and are indicated as potential biomarkers [7,31]. For example, ZFAS1 regulates miR-150-5p and by its influence on cellular phenotype. Another lncRNA, EGOT, is connected with HPV infection. Both of these lncRNAs could serve as prognostic biomarkers in HNSCC [32,33]. However, the exact function of the most lncRNAs is still unknown and needs to be further investigated. It is acknowledged that patients’ response to treatment depends on their unique cancer genetic pattern, which is manifested by its specific phenotype and behavior [34]. lncRNAs seem to be an important regulator of cancer cells and are promising therapeutic targets [15].

Treatment of locally advanced HNSCC includes surgical resection, radiotherapy, or radiochemotherapy, which may be preceded with induction chemotherapy. In recurrent or metastatic disease, the treatment is based on chemotherapy or immunotherapy [35]. However, the capacity to make a molecular-based decision about treatment strategy is limited [36].

The monitoring of patients’ response to the chosen therapeutic strategies and prediction of the probability of treatment response or disease progression during cancer therapy is one of the most important challenges in contemporary oncology [36,37]. One promising strategy is liquid biopsy, used to detect circulating nucleic acids, DNA or RNA, which are linked with specific patients’ condition and their response to treatment. This method could potentially also specify the survival prognosis of individuals [38]. The different types of RNAs, including protein-coding and non-coding molecules, include: miRNA, tRNA-derived RNA fragments (tRFs), circRNA, lncRNAs. All of these, and others, can be found in serum or plasma. Furthermore, they are present as free acids, complexes with proteins or lipids, or incorporated into extracellular vesicles, as for example into exosomes [39]. Many studies indicate the potential role of circulating miRNAs as biomarkers, but the role of other non-coding RNAs, such as lncRNAs, seems to be unclear and should be explored more thoroughly to establish the best possible biomarker panel [39].

In this study, based on the commercially available lncRNA qRT-PCR plate, the expression of 90 transcripts was examined in plasma samples of healthy volunteers and HNSCC patients. Next, the clinical usefulness of expression levels of plasma lncRNAs as a diagnostic and prognostic tool in locally advanced and palliative HNSCC patients was evaluated.

## 2. Materials and Methods

### 2.1. Patients and Volunteers

In this prospective analysis, 53 plasma samples from HNSCC patients and 14 healthy volunteers (18–80 years old) were obtained between April 2016 and May 2017. The control group consisted of 9 men and 5 women with no comorbidities. Seventeen naive HNSCC patients were treated with chemotherapy, induction chemotherapy first then followed by chemoradiotherapy or radiotherapy. The remaining 36 HNSCC patients were treated before our study, and later received palliative chemotherapy only. All of the examined patients were HPV (human papillomavirus) negative. The patients’ characteristics are shown in Supplementary Table S1.

### 2.2. Treatment

All patients in the induction group (locally advanced disease) were treated with a TPF regimen, which contained the following: docetaxel (T—75 mg/m^2^ day 1), cisplatin (P—75 mg/m^2^ day 1), and 5-fluorouracil (F—750 mg/m^2^ days 1–5). It was repeated three times during a 21 day period. Following computed tomography (CT), they were treated at the Radiotherapy Department. All patients in the palliative group (recurrent and/or metastatic disease) were treated using a PF regimen, which contained a cisplatin (P—100 mg/m^2^ day 1) and 5-fluorouracil (F—1000 mg/m^2^ days 1–5) regimen repeated three times during a 21 day period (Cetuximab was not reimbursed in Poland during this trial). After the treatment course of 3 cycles of chemotherapy, CT was performed and appropriate treatment was administered, but this aspect is beyond the scope of this study. When a response or stable disease were observed, in line with RECIST (Response Evaluation Criteria in Solid Tumors) 1.1 [40], the patients received up to 3 more cycles of PF. For patients with progressive disease (PD) according to RECIST 1.1, after the 3 cycles of chemotherapy mentioned above, a new chemotherapy regimen (Methotrexate 40 mg/m^2^ day 1 weekly) was administered. Prior to each therapy cycle, the patients were tested for adequate marrow, kidney and liver function. Additionally, a performance status was evaluated. The treatment response according to RECIST 1.1 depending on type of chemotherapy, induction or palliative, is presented in Supplementary Table S2.

### 2.3. Blood Sampling Design

Whole blood samples (5 mL) were taken from all patients receiving induction and palliative chemotherapy at two points in time: (i) before the start of treatment (P1), and (ii) after 3 cycles of chemotherapy (P2). In the volunteer group, blood samples were taken only once, namely in April 2016. All participating patients signed consent forms pertaining to the study’s aim, as well as personal and genetic data protection. The study methodologies conformed to the standards of the Declaration of Helsinki. The study did not violate the rights of other persons or institutions. The Bioethical Committee of Medical University of Poznan approved this study in agreement No. 152/2016.

### 2.4. Sample Collection

Pure blood samples were collected into tubes containing ethylenediaminetetraacetic acid (EDTA) (SARSTED, Monovette EDTA K) and immediately centrifuged (10 min; 3500 RPM, RT). The upper plasma phase was transferred to a new tube without disturbing the intermediate buffy coat layer and frozen at a temperature of −80 °C.

### 2.5. RNA Isolation

Before RNA isolation was conducted, all samples were checked for hemolysis using conventional macroscopic classification methods for samples [41]. The plasma samples were later thawed and only one cycle of thawing was conducted before taking 200 μL of material for further processing. The total RNA was isolated from plasma samples using miRNeasy Serum/Plasma Kit (Qiagen) according to the protocol for total RNA. A NanoDrop spectrophotometer (Thermo Scientific) was used to estimate the quality and quantity of the RNA samples, and then they were taken for further analysis, while the remaining RNA samples were stored at a temperature of –80 °C.

### 2.6. cDNA Synthesis and qRT-PCR Reaction

In the study, the 90 lncRNAs potentially connected with cancer and well-annotated in the lncRNA database (www.lncrnadb.org) were analyzed using the commercially available LncProfiler qPCR Array Kit (System Biosciences). The reaction consisted of three steps: (i) poly-A tailing, (ii) annealing anchor dT adaptor, (iii) complementary cDNA synthesis [42]. For the poly-A tailing step, 5 μL of RNA (about 100 ng) was used and mixed with 2 μL 5x PolyA Buffer, 1 μL MnCl_2_, 1.5 μL ATP and 0.5 μL polyA polymerase and incubated for 30 min at a temperature of 37 °C. Next, 0.5 μL of Oligo(dT) adapter was added and the reaction was heated for 5 min at 60 °C, and later cooled to room temperature. Finally, 4 μL of reverse transcription (RT) Buffer, 2 μL deoxynucleotide mix, 1.5 μL 0.1 M dithiothreitol (DTT), 1.5 μL Random Primer Mix and 1 μL reverse transcriptase were added and incubated for one hour at 42 °C, followed by 10 min at 95 °C.

cDNA was used for the qRT-PCR reaction using LightCycler 480 SYBR Green I Master buffer (Roche) and lncRNA primers from Primer Plate (component of the LncProfiler qPCR Array Kit) according to the manufacturer’s protocols.

The quantitative PCR reaction was performed using a preincubation step (95 °C for 10 min), 60 cycles of two-step amplification (95 °C for 15 s and 60 °C for 60 s) and a melting step with LightCycler 96 (Roche). All qRT-PCR data of 90 lncRNAs were analyzed by calculating the ΔC_t_, normalized against the mean expression of reference genes from the LncProfiler qPCR Array Kit (SBI), Supplementary Table S3. The fold-change in lncRNA expression was determined using the equation 2^−ΔCt^ and after that was compared to the appropriate group.

## 3. Statistical Analysis

Statistical analysis was performed with Jupyter Notebooks (5.7.2), Python (ver. 3.6), native libraries and ‘Lifelines’ library v.0.14.6 (Kaplan-Meier, COX) or GraphPad Prism 8. All data are presented as medians with 95% confidence intervals. Expression profiles of lncRNA were compared as follows: between HNSCC patients vs. healthy volunteers; HNSCC patients divided into two categories: (i) induction (locally advanced) vs. (ii) palliative treatment; localization of primary cancer lesion as well as treatment response in the palliative group were compared. The Shapiro–Wilk test was used to estimate data distribution, and, next, the T-test or Mann–Whitney tests were used to compare appropriate groups.

The correlation between clinical or laboratory parameters and lncRNA expression level was assessed using the Mann–Whitney and Kruskal–Wallis tests.

For diagnostic effectiveness of lncRNAs, ROC (receiver operating characteristic) calculation was performed and an optimal cut-off point was assessed according to the highest accuracy (minimal false negative and false positive rates). For prognostic purposes the value of AUC (area under the curve) of ROC for each lncRNA was calculated. For progression free survival (PFS) and overall survival (OS) in the palliative group, Kaplan–Meier curves were calculated.

The Cox proportional hazards regression model was used to determine which lncRNA is significantly associated with OS and PFS, and the results were presented as hazard ratios (HR) and 95% confidence intervals (CI). All of the statistical analyses were performed as two-tailed tests and considered significant at *p* < 0.05.

## 4. Results

### 4.1. Comparison of lncRNA Expression Levels between Healthy Volunteers and All HNSCC Patients

**Figure 1:**
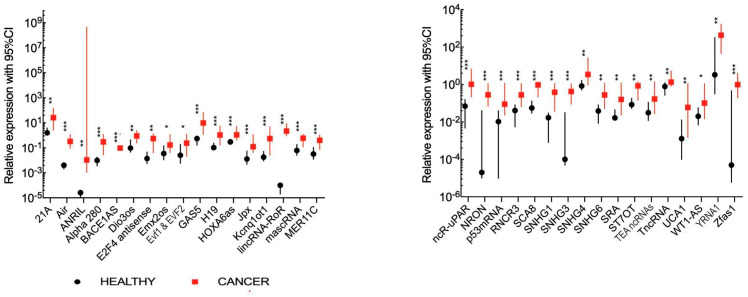
Significantly higher (*p* < 0.05) expression levels of 34 plasma lncRNAs between healthy volunteers (black) and head and neck squamous cell carcinoma (HNSCC) patients (red). Squares and circles represent means of expression and lines indicate 95% CI; * *p* < 0.05, ** *p* < 0.01, *** *p* < 0.001.

First, plasma lncRNA expression levels were compared in all HNSCC patients and all healthy volunteers. A significantly higher expression of 34 lncRNAs with a fold change of 0.49–2.1855 × 10^4^ was found in cancer patients. lncRNAs included: 21A, Air, ANRIL, Alpha 280, BACE1AS, Dio3os, E2F4 antisense, Emx2os, EvF1 and EVF2, GAS5, H19, HOXA6as, Jpx, Kcnq1ot1, lincRNA-RoR, mascRNA, MER11C, ncR-uPAR, NRON, p53 mRNA, RNCR3, SCA8, SNHG1, SNHG3, SNHG4, SNHG6, SRA, ST7OT, TEA ncRNAs, TncRNA, UCA1, WT1-AS, YRNA1 and Zfas1. All data are presented in Figure 1 and Supplementary Table S4. Next, ROC analysis was performed with an assessment of AUC for a prognostic value of these 34 selected lncRNAs. The differentiation between cancer patients and healthy individuals was possible with a sensitivity of 38.5–100% and a specificity of 57.1–100%. Details are presented in Supplementary Table S4.

### 4.2. Comparison of lncRNA Expression Levels between Healthy Volunteers and Recurrent and/or Metastatic HNSCC Patients

**Figure 2:**
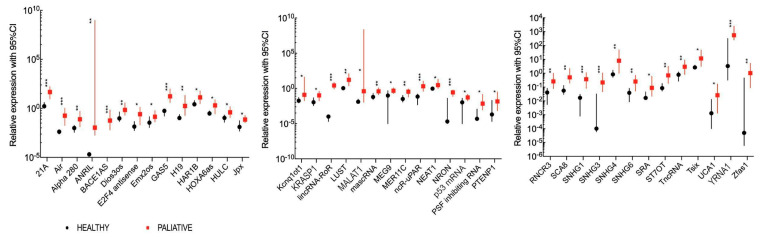
Significantly higher (*p* < 0.05) expression levels of 41 plasma lncRNAs between healthy volunteers (black) and recurrent and/or metastatic HNSCC patients (red). Squares and circles represent means of expression and lines indicate 95% CI; * *p* < 0.05, ** *p* < 0.01, *** *p* < 0.001.

The analysis revealed a significantly higher expression of 41 lncRNAs in recurrent and/or metastatic HNSCC patients compared to healthy volunteers with a fold change of 3.2–2.7 × 10^3^ for 21A, Air, Alpha 280, ANRIL, BACE1AS, Dios3os, E2F4 antisense, Emx2os, GAS5, H19, HAR1B, HOXA6as, HULC, Jpx, Kcnq1ot1, KRASP1, lincRNA-RoR, LUST, MALAT1, mascRNA, MEG9, MER11C, ncR-uPAR, NEAT1, NRON, p53 mRNA, PSF inhibiting RNA, PTENP1, RNCR3, SCA8, SNHG1, SNHG3, SNHG4, SNHG6, SRA, ST7OT, TncRNA, Tsix, UCA1, YRNA1 and Zfas1. All data are presented in Figure 2 and Supplementary Table S5. The ROC analysis with AUC estimation for prognostic value of 41 up-regulated lncRNAs was calculated. For the selected lncRNAs, the differentiation between studied groups was possible at a sensitivity and specificity between 38.9–100% and 42.9–100%, respectively (Supplementary Table S5).

### 4.3. Comparison of lncRNA Expression Levels between Healthy Volunteers and Locally Advanced HNSCC Patients

**Figure 3:**
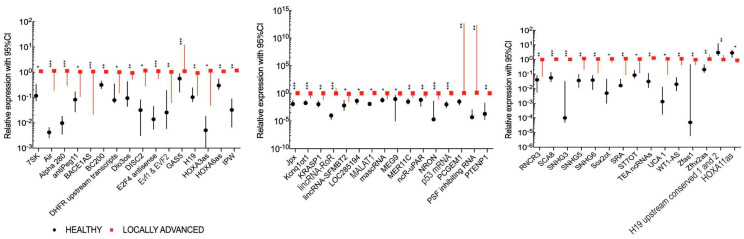
Significantly changed (*p* < 0.05) expression levels of 47 plasma lncRNAs between healthy volunteers (black) and locally advanced HNSCC patients (red). Squares and circles represent the mean of expression and lines indicate 95% CI; * *p* < 0.05, ** *p* < 0.01, *** *p* < 0.001.

In cancer patients, a significantly higher expression of 47 lncRNAs with fold change 1.96–5.101 × 10^4^ compared to healthy individuals was observed. However, a significantly lower expression of 2 lncRNAs (H19 upstream conserved 1 & 2, HOXA6as) with a fold change of 0.30–0.32 compared to healthy individuals was indicated, as shown in Figure 3 and Supplementary Table S6. The differentiation between study groups with high and low lncRNA expressions with ROC analysis was possible at a sensitivity and specificity of 37.5–100% and 71.4–100%, respectively. Details can be found in Supplementary Table S6.

### 4.4. Plasma lncRNA Expression Differs Depending on Primary Tumor Localization

**Figure 4:**
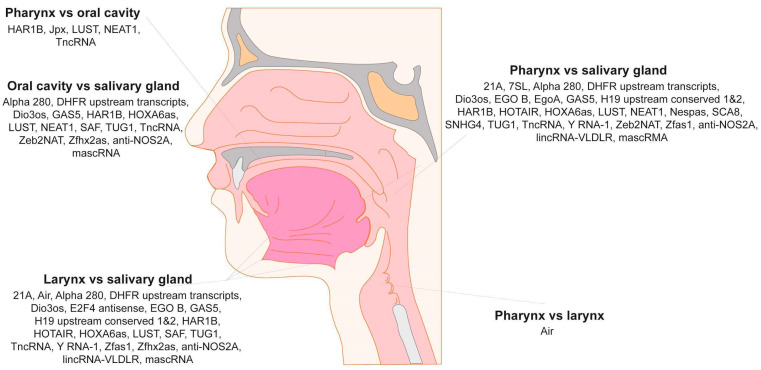
Changes in plasma lncRNA expression in HNSCC patients depending on the tumor localization in the pharynx, oral cavity, larynx and salivary gland sites. Only statistically significant changes (*p* < 0.05) in lncRNAs between groups are presented.

The association between the expression level of plasma lncRNAs and the primary site of the tumor was indicated. Five lncRNAs, HAR1B, Jpx, LUST, NEAT1 and TncRNA, showed significantly changed expressions when pharynx and oral cavity localizations were compared. For cancers with pharyngeal and laryngeal localizations, lncRNA Air was only significantly altered. The most frequent changes in lncRNA expressions were detected between patients with salivary gland cancers in comparison to other tumor sites. All data are presented in Figure 4 and Supplementary Table S7.

### 4.5. Comparison of lncRNA Expression Levels According to Treatment Response in HNSCC Patients Receiving Palliative Chemotherapy

**Figure 5:**
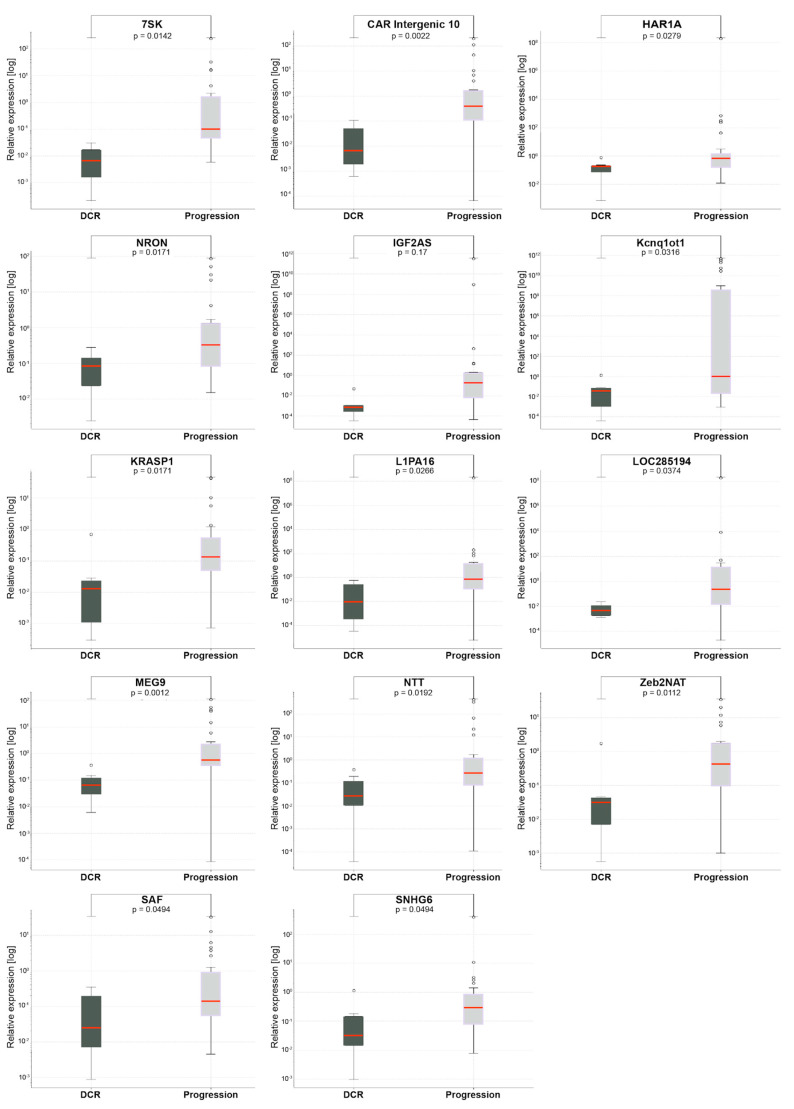
lncRNA expressions in HNSCC patients receiving palliative chemotherapy according to response to treatment (disease control rate vs. progression). Data present median expression of lncRNA with confidence interval (CI); *p* < 0.05.

The different responses of the studied patients to treatment were assessed according to RECIST 1.1 criteria (Supplementary Table S2). Additionally, lncRNA expressions in specific subgroups were analyzed. In HNSCC, in the group receiving palliative chemotherapy, patients with PD (progressive disease) showed significantly high expressions of 14 lncRNAs compared to patients with DCR (disease control rate, CR+PR+SD), 7SK (*p* = 0.0142), CAR Intergenic 10 (*p* = 0.0022), HAR1A (*p* = 0.0279), NOR (*p* = 0.0171), IGF2AS (*p* = 0.017), Kcnq1ot1 (*p* = 0.0316), KRASP1 (*p* = 0.0171), L1PA16 (*p* = 0.0266), LOC285194 (*p* = 0.0374), MEG (*p* = 0.0012), NTT (*p* = 0.0192), Zeb2NAT (*p* = 0.0112), SAF (*p* = 0.0494), and SNHG6 (*p* = 0.0494), as shown in Figure 5. All patients responded to induction chemotherapy. Accordingly, it was not possible to conduct a comparative study.

### 4.6. Changes in lncRNA Expression Levels during Induction Chemotherapy and Palliative Chemotherapy

**Figure 6:**
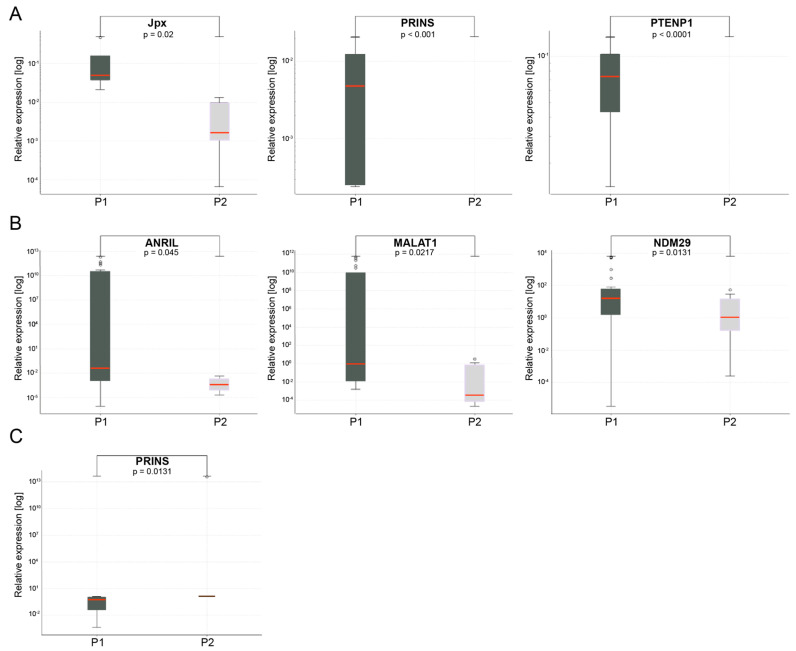
lncRNA expression in palliative group with (**A**) disease control rate (DCR) and (**B**) progressive disease (PD) response, and (**C**) in a locally advanced group, depending on two time points—before chemotherapy (P1) and after three cycles of chemotherapy (P2). Data present median expression of lncRNA with confidence interval (CI); *p* < 0.05.

Subsequent analysis of changes in lncRNA expressions in a palliative group of eight patients with DCR at two time points—before treatment (P1) and after three cycles of chemotherapy (P2)—was performed. Significant downregulation of three lncRNAs, Jpx (*p* = 0.02), PRINS (*p* < 0.001) and PTENP1 (*p* < 0.0001), was observed, as shown in Figure 6A. In the remaining 28 patients included in the palliative group, a progression of disease (PD) was observed. In these patients, statistically significant downregulation of lncRNAs ANRIL (*p* = 0.045), MALAT1 (*p* = 0.0217) and NDM29 (*p* = 0.043) between two time points—(P1) and (P2)—was found, as shown in Figure 6B.

All patients (n = 17) with locally advanced HNSCC treated with induction chemotherapy presented a DCR response during this study. Analysis of lncRNA expressions in these patients revealed that only PRINS was significantly downregulated (*p* = 0.0131) after three cycles of chemotherapy (P2) compared to pretreatment (P1) levels (Figure 6C).

### 4.7. Assessment of lncRNA Expression in HNSCC Patients Receiving Palliative Chemotherapy Can Serve as a Prognostic Biomarker

Patients included in the palliative group were divided into two subgroups with high or low expressions of each lncRNA according to the mean expression level as a cut-off. Moreover, Kaplan–Meier curves were calculated for OS and PFS for each of the 90 examined lncRNAs.

**Figure 7:**
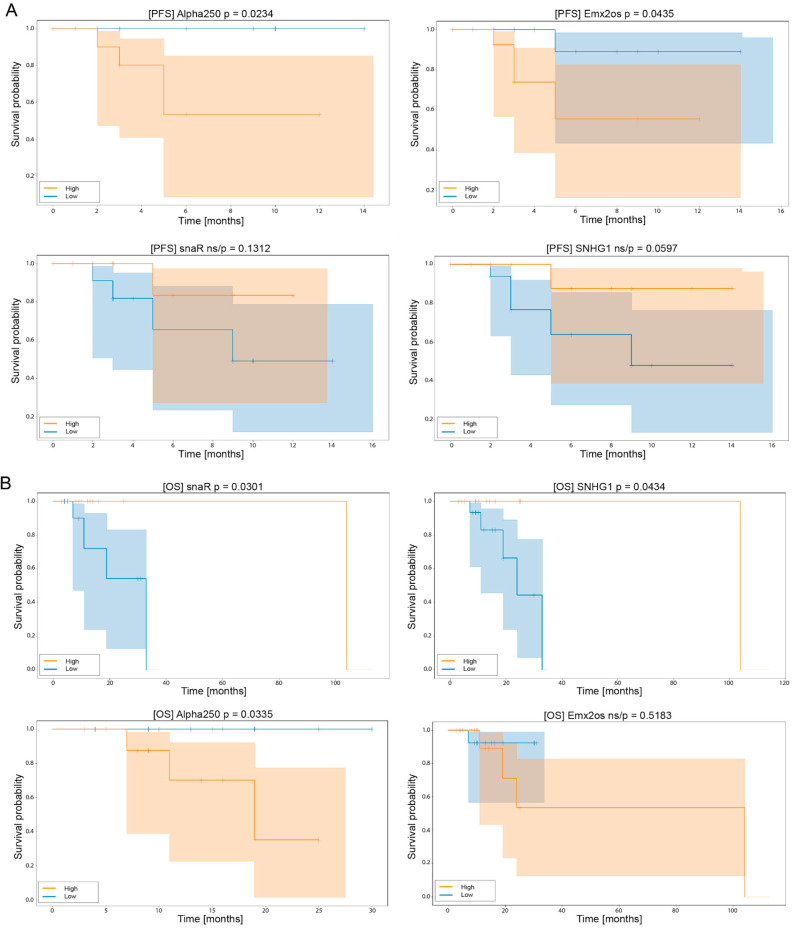
Progression free survival probability (**A**) and overall survival probability (**B**) of HNSCC patients receiving palliative chemotherapy depending on expression levels of Alpha 250, Emx2os, snaR and SNHG1 lncRNAs. Patients were divided into two subgroups according to high (red)/low (blue) expression levels of lncRNA, Kaplan–Meier analysis, and *p* < 0.05 considered as statistically significant.

Two of the lncRNAs, Alpha 250 and Emx2os, manifested a statistically significant difference in PFS in respect to high/low levels of expression. For patients with high expressions of Alpha 250 or Emx2os, a five month (95% CI (0.4–0.9) and 95% CI (0.1–0.9), respectively) median of PFS was noticed. Moreover, median PFS was not reached in the group with low expressions of these lncRNAs, and patients were characterized by a longer time without progression, as shown in Figure 7A.

Next, the OS in respect to high/low expression levels of all lncRNAs was examined, and three of them, snaR, SNHG1 and Alpha 250, showed statistically significant differences between groups of patients. In patients with low expressions of snaR or SNHG1, the survival of patients was shorter compared to the group with higher expressions of these lncRNAs: 30 month (95% CI (0.15–0.8), *p* = 0.03) and 22 month (95% CI (0.2–0.8), *p* = 0.043) mean of OS was respectively observed. In both cases, a median OS was not reached in patients with high expressions.

In the group of patients with high expressions of Alpha 250, a 17 month median OS was noticed (95% CI (0.2–0.9), *p* = 0.033). In the low expression group, the median was not reached, and patients revealed a longer OS compared to the group with high expression levels of this lncRNA, as shown in Figure 7B.

For further analysis, univariate and multivariate Cox regression models were performed to assess the independence of the prognostic value of Alpha 250, snaR and Emx2os, which were later indicated using Kaplan–Meier curves as prognostic factors. Univariate and multivariate models showed that Alpha 250 is only a statistically significant factor for probability of OS and PFS, and it is independent of age, neutrophil to lymphocyte ratio (NLR), body mass index (BMI) and primary localization of tumor. All data are presented in Table 1 and Table 2.

### 4.8. lncRNA Expression Levels and Clinical and Laboratory Parameters of HNSCC Patients

Expression levels of Alpha 250, Emx2os, snaR and SNHG1 were analyzed in terms of clinical and laboratory parameters that may be helpful in the assessment of patients’ prognosis. Significant differences between stage IVA and IVC for SNHG1 (0.0823 vs. 0.4601, *p* = 0.0401) and snaR in younger patients compared to older ones (2.514 vs. 12.1099, *p* = 0.0183) were observed. Regarding Alpha 250 and Emx2os, no significant differences in expression levels within the group of patients divided by age, sex, body mass index, nicotinism, alcoholism, localization of primary tumor, disease stage, neutrophil to lymphocyte ratio and platelets to lymphocyte ratio were noticed. All data are presented in Table 3.

## 5. Discussion

Approximately half of HNSCC cases are diagnosed in an advanced stage with poor outcome [43]. Accordingly, the identification of low invasive and simple biomarkers for these patients’ prognosis assessment is crucial. Recently, an intensive search, including liquid biopsy based on circulating nucleic acids, such as DNA and RNA [38,39,44], has been carried out. It was previously indicated that lncRNAs can be found in tumor tissue (including HNSCC) and in bodily fluids [44,45]. However, circulating lncRNA as potentially predictive and prognostic biomarkers in advanced or metastatic HNSCC were not reported.

The first important finding is that the level of plasma lncRNA expressions in HNSCC patients and healthy volunteers differs significantly. In addition, it directly correlates with the stage of the disease. Out of the 90 lncRNAs studied, 47 were high up- and 2 down-regulated in patients with locally advanced cancers. A total of 41 lncRNAs were up-regulated in patients with recurrent and/or metastatic cases, while 29 lncRNAs were down-regulated in locally advanced and recurrent and/or metastatic HNSCC. Altered lncRNA expressions enabled differentiation of HNSCC patients from a healthy population with high sensitivity and specificity, proving lncRNA to be a good biomarker. Yao et al. demonstrated the expression of lncRNAs in plasma and in cancer tissue using microarray and next-generation RNA-sequencing methods (NGS) validated by qRT-PCR. They reported changes in 432 lncRNA transcripts in plasma and 333 in tissue (fold changes > 4). However, the expression of only three lncRNA in the plasma of HNSCC patients was significantly higher than in healthy controls (HOXA11as, LINC00964 and MALAT1) and might be used as potential circulating biomarkers for early detection [45]. In our study, we observed changes in 34 out of 90 examined lncRNAs. In contrast to Yao et al., we did not observe expression changes in HOXA11as, LINC00964 and MALAT1 in all groups of HNSCC patients compared with healthy donors. However, up-regulation of HOXA11as and MALAT1 in patients with locally advanced and recurrent and/or metastatic HNSCC was indicated. Interesting, we observed multiple expression changes in lncRNAs associated with HOXA (homeobox A), a cluster referred to as HOXA3as, HOXA6as and HOXA11as, as well as with lncRNAs associated with SNHGs (small nucleolar RNA host genes) such as SNHG1, SNHG3, SNHG4, SNHG5 and SNHG6. However, until now there are no data to point to the role of HOXA3as and HOXA6as in cancer. Only HOXA11as is indicated as an important lncRNA in progression and metastasis of a number of different cancers by regulating proteins and miRNAs crucial in cellular processes such as cell cycle, apoptosis, epithelial-to-mesenchymal transition (EMT) process, invasion and metastasis [46,47]. SNHG1 was also described as an important modulator of cancer progression [48]. In laryngeal squamous cell carcinoma (LSCC) the expression of SNGH1 was up-regulated, connected with cell proliferation, EMT process, metastasis and poor patients’ prognosis [49]. SNHG1 inhibits LSCC growth and metastasis in vitro by promoting YAP1 expression and Hippo signaling activity through sponging miR-375 [50]. Wang et al. indicated that SNHG3 regulates the proliferation and migration of LSCC by way of modulation within the miR-384/WEE1 axis [51]. SNHG4 is not described for HNSCC, but in lung cancers it affects proliferation, migration and invasiveness, as well as EMT through miR-98-5p [52]. Similarly, SNHG5 in nasopharyngeal carcinoma regulates the expression of both the miR-1179/HMGB3 axis and cancer progression [53]. Finally, SNHG6 was indicated in the meta-analysis as an oncogene connected with cancer progression and could be used as a promising prognostic biomarker [54]. Only two studies described its role in HNSCC. It was shown that SNHG6 was up-regulated in tongue cancer, and its knockdown inhibited cell viability and proliferation, induced apoptosis and prevented EMT in vitro [55]. Guo et al., observed the potential role of SNHG6 in the oncogenesis of HPV-positive oropharyngeal squamous cell carcinoma (OPSCC) [56]. Moreover, we found up-regulation of Y RNA-1 (RNY1) transcripts in our HNSCC patients. Our observation confirmed a previous report by Victoria Martinez et al., who, using NGS, demonstrated changes in Y RNAs transcripts in serum from HNSCC patients as compared to healthy donors [57]. In our previous study, we demonstrated that in BRAF-mutant metastatic melanoma patients treated with a BRAF inhibitor, a higher expression of Y RNA-1 was associated with worse prognosis [58]. Furthermore, Guglas et al., based on HNSCC cell lines, patient samples and TCGA data, indicated that Y RNA-1 was significantly down-regulated, while its higher expression was associated with worse patient survival [59]. More and more data indicate that YRNAs as well as YRNA-derived fragments are important regulators in tumorigenesis, and they have a potential role in diagnostics [60].

In the group of locally advanced HNSCC patients, two lncRNAs were downregulated compared to healthy volunteers, described above HOXA6as, and H19 upstream conserved 1 and 2. Drewell et al. indicated that conserved elements upstream of the H19 gene are transcribed and act as mesodermal enhancers [61]. There is no information about H19 upstream-conserved 1 and 2, nor about its role in the context of circulating transcripts and immune cells.

Unfortunately, we have not elucidated the nature of the observed lncRNA expression alterations and the potential function of them. Still, the origin of circulating lncRNA is unclear, and some evidence suggests it could be tumor tissue, tumor cells, something related to the immune system or even blood cells [62,63]. Yao et al., indicated that only 12 lncRNA expressions are changed in the plasma and tissue of HNSCC patients [45]. This suggests the remaining changed lncRNAs could demonstrate different origins, but this requires more studies. We also observed, like others, that the expression of lncRNAs is changed depending on the patient’s status. Differences between lncRNA expression levels between cancer patients and healthy individuals, and in two patient groups, are probably due to changes in physiognomy during cancer progression, earlier treatment of metastatic disease and treatment resistance, as well as the stage of the disease. The relatively small group included in the study may also be a possible explanation of the results obtained.

Our comparison of primary tumor localization and lncRNA expression indicated some similarities, as well as differences in transcript expressions. In salivary glands, different lncRNA expressions were reported as in other tumors. This might be due to the different etiology of the salivary glands and larynx, pharynx and oral cavity cancers, which is mainly due to alcohol abuse and nicotinism [64].

An important finding of our study shows the lncRNA expression pattern in a metastatic group of patients with primary resistance to treatment. Reports about circulating lncRNAs and resistance to therapy are limited, and, to the best of our knowledge, this is the first identification of 14 up-regulated lncRNAs (7SK, CAR intergenic 10, HAR1A, IGF2AS, Kcnq1ot1, KRASP1, L1PA16, LOC285194, MEG9, NTT, NRON, Zeb2NAT, SAF, SNHG6). Moreover, these RNAs could probably be used as biomarkers of primary resistance in HNSCC. Furthermore, during palliative chemotherapy, reduction of Jpx, PRINS and PTENP1 expression levels after three treatment cycles correlated with treatment response. In contrast, the reduction of lncRNAs named ANRIL, MALAT1 and NDM29 was reported in these patients while developing progressive disease. All patients treated with induction chemotherapy responded to treatment, and these patients have up-regulated PRINS measured during the second blood collection. It is possible that the aforementioned effect was related to the treatment applied.

It is difficult to compare our results with other studies since there are no similar reports available. Only one study examined the potential role of plasma lincRNA-p21, GAS5 and HOTAIR as biomarkers to predict a response to chemoradiotherapy, which demonstrated that only GAS5 was useful when measured after chemotherapy [65]. In contrast, our study did not confirm these results. However, we observed significant differences in expression levels of GAS5 between HNSCC patients and healthy volunteers, as well as between HNSCC patients—locally advanced and recurrent and/or metastatic disease compared to non-cancer.

Most studies of chemical agents on lncRNA expressions were based on in vitro models using cell lines. Guglas et al. based their study on HNSCC cell lines (SCC-040, SCC-25, FaDu and Cal27) observed changes in lncRNA expressions after exposure to irradiation and chemotherapeutic drugs. These changes were dose (5, 10 or 20 Gy) and type (cisplatin or doxorubicin) dependent, respectively. Cisplatin down-regulated CAR Intergenic 10, PRINS, and PTENP1 expressions, while doxorubicin down-regulated MALAT1 and others in surviving cells, compared to those non-treated with toxic agents [25]. Another study indicated that ANRIL, HOTAIR and MALAT1 were significantly down-regulated in cancer tissue compared to adjacent normal tissues. Moreover, expressions of these lncRNAs were reduced in laryngeal cell lines (Hep-2 and AMC-HN8) after exposition to cisplatin and paclitaxel [66]. Ren et al., using NGS, compared two nasopharyngeal cell lines—parental CNE-2 and paclitaxel-resistant—and revealed 2670 known and 4820 novel lncRNAs between them. From the altered lncRNAs, they selected the n375709 transcript, and its knock-down in the NPC 5–8F and 6–10B cell lines increased cells’ sensitivity to paclitaxel in vitro [67]. The regulation of chemosensitivity in nasopharyngeal cancers also displays lncRNA-ROR. It is up-regulated in NPC tissue as well as cell lines and affects cell proliferation, cell cycle, apoptosis, migration, and invasion. In vitro studies indicated that lncRNA-ROR regulates the EMT process. Moreover, cis-dichlorodiammineplatinum (II) (DDP) is induced in the cell line expression of lncRNA-ROR. Artificial down-regulation of lncRNA-ROR is caused by DDP influence on the p53 pathway [68].

Clinical and laboratory parameters showed a statistically significant correlation between low expressions of SNHG1 and stage IVA of disease. The expression of snaR was associated with a younger age in patients as well. It is difficult to conclude these findings, as there is a lack of information on lncRNA expression levels based on the stage of disease and age of patients in the literature.

Our final important finding is the observation that patients with down-regulated Alpha 250 and Emx2os display longer PFS compared to those with upregulation. In addition, the down-regulation of Alpha 250 was favorable also for longer OS. It is the first report of Alpha 250 in the context of cancer and HNSCC. The origin of Alpha 250 and Alpha 280 transcripts is the first intron of the *Rps14* gene, which encodes S14 proteins, a part of the 40S ribosome subunit responsible for the translation and synthesis of proteins. Up-regulated expressions of Alpha 250 are linked with cell proliferation [69]. Tang et al. identified that low expressions of Emx2os are closely associated with longer DSF and OS ratio in LSCC patients based on in silico analysis of databases. They also indicated that Emx2os regulates the expression of CALCA and GABRG2 through miR-124 [70]. We have also observed that high expressions of snaR and SNHG1 was correlated with a favorable OS in HNSCC patients. Liang et al. showed that an overexpression of plasma snaR is correlated with poor OS in LSCC patients and positively correlated with TGF-β1. Moreover, snaR overexpression coupled with TGF-β1 treatment promoted cell proliferation, migration, and invasion in vitro [71]. SNHG1 was indicated also in LSCC but, as mentioned above, not only in tissue and cell lines but also as circulating ncRNAs. Its higher expression was connected with poorer OS [49,50]. However, our study based on profiling of lncRNA expression in melanoma patients indicated that a low expression of SNHG1 seems to be a good marker of DSF [58].

Univariate and multivariate Cox regression models for selected lncRNAs indicated that only plasma Alpha 250 proved to be a prognostic biomarker, and it is the first time this biomarker for chemotherapy resistance in plasma was identified.

## 6. Conclusions, Limitation and Future Perspective

In this study, we examined the expression profile of 90 different lncRNAs in plasma taken from 53 HNSCC patients (17 with locally advanced and 36 with metastatic disease) during chemotherapy and from 14 healthy volunteers. Based on these results, there are four major findings in our study: (i) plasma lncRNAs in HNSCC patients are markers for disease diagnosis; (ii) plasma lncRNA expression levels may serve as biomarkers of primary chemotherapy resistance in HNSCC patients; (iii) lncRNA expression levels bear prognostic biomarker potential for treatment efficacy; and (iv) lncRNA Alpha 250 is a prognostic biomarker for OS and PFS of HNSCC patients.

As observed, the plasma-based liquid biopsy and quantification of lncRNAs is a potentially clinically useful tool for the evaluation of locally advanced and metastatic HNSCC patients. However, our study is one of the first to describe plasma based lncRNAs in this specific group of patients, and it is difficult to discuss the results in a broader perspective. The lack of similar data for lncRNA Alpha 250, as well as for the most of indicated here lncRNAs in the context of HNSCC, makes our study limited. To verify our results, an independent study based on the large group of locally advanced and metastatic HNSCC patients should be undertaken.

The second important question is the source of the circulating transcripts. Unfortunately, we did not examine the tissue from analyzed patients, so it is difficult to answer if the observed lncRNAs are released from cancer tissue to the bloodstream or are a whole body response to the disease and treatment strategy. In the future, studies like these should be more comprehensive and profiling of transcripts in cancer tissue and circulating need to be obligatory. Moreover, future functional studies about the potential role of circulating lncRNAs could give a more comprehensive point of view in relation to whether these transcripts influence the potential regulatory role in other cells such as immune cells, or are only a consequence of still unknown processes. However, when it comes to biomarkers, the aforementioned questions are not so important, because their principal role is the description of the state of the patient and they provide clinical answers about a current or a potential treatment strategy.

## Figures and Tables

**Table 1:** Univariate Cox regression model for PFS and OS. PFS—progression free survival; OS—overall survival; HR—hazard ratio; CI—confidence interval; BMI—body mass index; NLR—neutrophil to lymphocyte ratio.

Parameter	Group Stratification	PFS			OS		
		*p*-Val	HR	95% CI	*p*-Val	HR	95% CI
Age	<61 vs. >61	0.82	0.80	0.16–4.2	0.30	0.41	0.06–2.61
NLR	<5 vs. >5	0.54	0.60	0.12–3.06	0.90	0.51	0.19–6.25
BMI	<18.49 vs. >25	0.89	1.10	0.22–5.75	0.46	2.15	0.28–16.8
Primary tumor localization	Pharynx vs. oral cavity	0.40	0.67	0.01–30.1	0.44	0.23	0.0–10.48
Primary tumor localization	Pharynx vs. larynx	0.40	0.67	0.01–30.1	0.44	0.23	0.0–10.48
Primary tumor localization	Pharynx vs. salivary gland	0.40	0.67	0.01–30.1	0.44	0.23	0.0–10.48
Primary tumor localization	Oral cavity vs. salivary gland	0.46	1.80	0.36–9.27	0.44	0.22	0.14–8.60
Primary tumor localization	Larynx vs. salivary gland	0.90	1.10	0.21–5.76	0.90	1.09	0.14–8.60
lncRNA Alpha 250	High vs. low expression	**0.039**	0.10	0.01–0.89	**0.042**	0.20	0.01–0.88
lncRNA Emx2os	High vs. low expression	0.13	0.26	0.04–1.54	-	-	-
lncRNA snaR	High vs. low expression	-	-	-	0.20	3.0	0.45–20.50
lncRNA SNHG1	High vs. low expression	-	-	-	0.10	6.9	0.66–74.12

**Table 2:** Multivariate Cox regression model for PFS and OS. PFS—progression free survival; OS—overall survival; HR—hazard ratio; CI—confidence interval; BMI—body mass index; NLR—neutrophil to lymphocyte ratio.

Parameter	Group Stratification	PFS			OS		
		*p*-Val	HR	95%CI	*p*-Val	HR	95%CI
Age	<61 vs. >61	0.90	0.84	0.02–30.31	0.60	0.48	0.03–9.05
NLR	<5 vs. >5	0.90	0.86	0.02–33.56	0.90	0.80	0.06–12.33
BMI	<18.49 vs. >25	0.90	1.09	0.22–47.52	0.90	1.01	0.02–43.42
Primary tumor localization	Pharynx vs. oral cavity	0.90	0.91	0–285.89	0.80	0.23	0–135.53
Primary tumor localization	Pharynx vs. larynx	0.90	0.91	0–285.89	0.80	0.69	0–135.53
Primary tumor localization	Pharynx vs. salivary gland	0.90	0.91	0–285.89	0.80	0.60	0–135.53
Primary tumor localization	Oral cavity vs. salivary gland	0.80	1.48	0.04–55.74	0.90	0.80	0.04–19.84
Primary tumor localization	Larynx vs. salivary gland	0.90	0.93	0.2–37.21	0.70	1.73	0.07–41.21
lncRNA Alpha 250	High vs. low expression	**0.039**	0.20	0.01–0.87	**0.043**	0.30	0.02–0.89
lncRNA Emx2os	High vs. low expression	0.80	0.67	0.02–24.62	-	-	-
lncRNA snaR	High vs. low expression	-	-	-	0.40	3.07	0.17–55.67
lncRNA SNHG1	high vs. low expression	-	-	-	0.50	2.77	0.14–54.37

**Table 3:** Expression levels of Alpha 250, Emx2os, snaR and SNHG1 lncRNAs depending on clinical and laboratory parameters in HNSCC patients; N—BMI between 18.5–24,8; PN—BMI under 18.5, BMI—body mass index, NLR—neutrophil to lymphocyte ratio, PLR—platelets to lymphocyte ratio. Mann–Whitney test and one-way ANOVA; *p* < 0.05 considered as statistically significant.

Parameter	Alpha 250				Emx2os				snaR				SNHG1			
	Median	Lower Quartile	Upper Quartile	*p*-Val	Median	Lower Quartile	Upper Quartile	*p*-Val	Median	Lower Quartile	Upper Quartile	*p*-Val	Median	Lower Quartile	Upper Quartile	*p*-Val
Age																
>61	0.0035	0.0014	0.0826	0.6525	0.2418	0.0257	0.5946	0.6525	12.1099	7.024	26.7425	**0.0183**	0.5092	0.1419	1.3947	0.3046
<61	0.0679	0.007	1.2219		0.0804	0.0462	0.3803		2.514	0.7354	5.9042		0.2833	0.037	1.1563	
Sex																
Female	73.826	0.0932	155.012	0.7974	0.1554	0.0352	40.359	0.9071	12.261	0.4948	930.9114	0.6916	0.348	0.0224	43.1257	0.7616
Male	0.0095	0.0026	0.1151		0.1181	0.0458	0.5864		4.7372	1.2829	17.1813		0.4133	0.1042	1.1313	
Nicotinism																
Yes	0.0446	0.0072	0.6322	0.2826	0.484	0.0473	1.1299	0.7293	10.3091	1.8426	31.6433	0.4775	0.6552	0.3728	1.16	0.1216
No	0.0065	0.0024	0.2138		0.1031	0.0311	0.2635		2.7132	0.3886	10.6872		0.1672	0.0715	0.6246	
Alcoholism																
Yes	0.1486	0.0089	0.9224	0.8107	0.2418	0.0518	2.3202	0.6718	5.5867	1.8152	25.3311	0.6451	0.3728	0.0641	1.1551	0.5932
No	0.0035	0.002	0.0396		0.1106	0.0348	0.5187		5.5404	0.6406	12.1099		0.4601	0.0881	1.5281	
BMI																
N	0.024	0.0029	0.1724	0.5619	0.2132	0.0907	0.4404	0.9255	3.934	2.0813	12.261	0.779	0.4756	0.139	1.4659	0.779
PN	0.0099	0.0024	0.532		0.0595	0.0284	0.8953		7.0999	0.844	22.5037		0.3253	0.0739	1.0851	
NLR 1																
>5	0.1254	0.0055	0.4669	0.2862	0.2418	0.0518	0.3917	0.2701	4.4906	0.6604	13.9262	0.8708	0.3524	0.045	0.9596	0.7863
<5	0.0067	0.001	0.0772		0.1022	0.0247	0.5926		5.5404	1.5491	29.7874		0.4298	0.1075	2.0887	
PLR 1																
>215	0.0399	0.0032	0.2521	0.4602	0.2635	0.0555	0.7589	0.1886	2.4156	0.8338	11.3605	0.3775	0.2184	0.0565	0.7585	0.3219
<215	0.0098	0.0018	0.2011		0.0804	0.0298	0.5809		9.1135	2.514	35.8773		0.5257	0.148	2.425	
Stage																
IVA	0.0026	0.0005	0.0035	0.215	0.1031	0.0257	0.5864	0.7916	0.976	0.2823	9.1135	0.1378	0.0823	0.0213	0.4832	**0.0401**
IVC	0.0679	0.0093	0.3368		0.1629	0.0458	0.5763		6.268	2.1179	23.6976		0.4601	0.1672	1.1551	
Localization																
Pharynx	0.2011	0.1021	0.4869	0.245	0.2465	0.076	0.557	0.6652	3.934	2.3602	17.083	0.4077	0.3994	0.2391	0.7773	0.5987
Larynx	0.0293	0.0037	0.2107		0.0518	0.0341	0.2938		3.8109	1.1966	9.4315		0.2778	0.0773	0.8421	
Salivary gland	0.0001	0.0001	0.0026		0.3061	0.0177	11.5981		69.1904	23.6976	81.0084		2.1747	0.4275	33.5545	
Oral cavity	0.025	0.0082	0.2494		0.1908	0.0704	0.59		2.7132	1.3064	10.5361		0.2972	0.0739	0.7585	

## Data Availability

The datasets used and/or analyzed during the current study are available from the corresponding author on reasonable request.

## Supplementary Materials

The following are available online at https://www.mdpi.com/2075-4426/10/3/131/s1, Table S1: Baseline characteristic of patients included into the study. Table S2: Treatment response as defined by RECIST 1.1. Table S3: Examined lncRNAs and reference genes ncProfiler qPCR Array Kit (System Biosciences). Table S4: Median expression of lncRNA in healthy volunteers and HNSCC patients with Confidence Interval (CI), and prognostic value of lncRNAs with sensitivity and specificity features to distinguish the examined groups. Table S5: Median expression of lncRNA in volunteers and palliative treated HNSCC patients with Confidence Interval (CI), and prognostic value of lncRNAs with sensitivity and specificity features to distinguish the examined groups. Table S6: Median expression of lncRNA in volunteers and locally advanced HNSCC patients with Confidence Interval (CI), and prognostic value of lncRNAs with sensitivity and specificity features to distinguish the examined groups. Table S7: lncRNA expression according to the primary site of tumor.

## References

[1] Shield K.D., Ferlay F., Jemal A., Sankaranarayanan R., Chaturvedi A.K., Bray F., Soerjomataram I. (2017). The global incidence of lip, oral cavity, and pharyngeal cancers by subsite in 2012. CA Cancer J. Clin..

[2] Tsao S.W., Tsang C.M., Lo K.W. (2017). Epstein-Barr virus infection and nasopharyngeal carcinoma. Philos. Trans. R. Soc. Lond. B Biol. Sci..

[3] The Cancer Genome Atlas Network (2015). Comprehensive genomic characterization of head and neck squamous cell carcinomas. Nature.

[4] Tonella L., Giannoccaro M., Alfieri S., Canevari S., De Cecco L. (2017). Gene Expression Signatures for Head and Neck Cancer Patient Stratification: Are Results Ready for Clinical Application?. Curr. Treat. Options Oncol..

[5] Solomon B., Young R.J., Rischin D. (2018). Head and neck squamous cell carcinoma: Genomics and emerging biomarkers for immunomodulatory cancer treatments. Semin. Cancer Biol..

[6] Sannigrahi M.K., Sharma R., Panda N.K., Khullar M. (2018). Role of non-coding RNAs in head and neck squamous cell carcinoma: A narrative review. Oral Dis..

[7] Guglas K., Bogaczyńska M., Kolenda T., Ryś M., Teresiak A., Bliźniak R., Łasińska I., Mackiewicz J., Lamperska K. (2017). lncRNA in HNSCC: Challenges and potential. Contemp. Oncol..

[8] Arantes L.M.R.B., De Carvalho A.C., Melendez M.E., Carvalho A.L. (2018). Serum, plasma and saliva biomarkers for head and neck cancer. Expert Rev. Mol. Diagn..

[9] Orgel L.E., Crick F.H.C. (1980). Selfish DNA: The ultimate parasite. Nature.

[10] Niu D.K., Jiang L. (2013). Can ENCODE tell us how much junk DNA we carry in our genome?. Biochem. Biophys. Res. Commun..

[11] Kiss T. (2004). Biogenesis of small nuclear RNPs. J. Cell Sci..

[12] Prensner J.R., Chinnaiyan A.M. (2011). The emergence of lncRNAs in cancer biology. Cancer Discov..

[13] Gomes A.Q., Nolasco S., Soares H. (2013). Non-Coding RNAs: Multi-Tasking Molecules in the Cell. Int. J. Mol. Sci..

[14] Chi Y., Wang D., Yu W., Yang J. (2019). Long Non-Coding RNA in the Pathogenesis of Cancers. Cells.

[15] Schmitt A.M., Chang H.Y. (2016). Long Noncoding RNAs in Cancer Pathways. Cancer Cell.

[16] Yao R.W., Wang Y., Chen L.L. (2019). Cellular functions of long noncoding RNAs. Nat. Cell Biol..

[17] Derrien T., Johnson R., Bussotti G., Tanzer A., Djebali S., Tilgner H., Guernec G., Martín D., Merkel A., Knowles D.G. (2012). The GENCODE v7 catalog of human long noncoding RNAs: Analysis of their gene structure, evolution, and expression. Genome Res..

[18] Johnsson P., Lipovich L., Grandér D., Morris K.V. (2014). Evolutionary conservation of long non-coding RNAs; sequence, structure, function. Biochim. Biophys. Acta.

[19] Hubbard T., Barker D., Birney E., Cameron G., Chen Y., Clark L., Cox T., Cuff J., Curwen V., Down T. (2002). The Ensembl genome database project. Nucl. Acid Res..

[20] Morris K.V., Mattick J.S. (2014). The rise of regulatory RNA. Nat. Rev. Genet..

[21] Iyer M.K., Niknafs Y.S., Malik R. (2015). The landscape of long noncoding RNAs in the human transcriptome. Nat. Genet..

[22] Ji Z., Song R., Regev A., Struhl K. (2015). Many lncRNAs, 5′UTRs and pseudogenes are translated and some are likely to Express functional proteins. eLife.

[23] Yan L., Yang M., Guo H., Yang L., Wu J., Li R., Liu P., Lian Y., Zheng X., Yan J. (2013). Single-cell RNA-Seq profliling of human preimplantation embryos and embryonic stem cells. Nat. Struct. Mol. Biol..

[24] Pirogov S.A., Gvozdev V.A., Klenov M.S. (2019). Long Noncoding RNAs and Stress Response in the Nucleolus. Cells.

[25] Guglas K., Kolenda T., Teresiak A., Kopczyńska M., Łasińska I., Mackiewicz J., Mackiewicz A., Lamperska K. (2018). lncRNA Expression after Irradiation and Chemoexposure of HNSCC Cell Lines. Noncoding RNA.

[26] Puvvula P.K. (2019). LncRNAs Regulatory Networks in Cellular Senescence. Int. J. Mol. Sci..

[27] Dahariya S., Paddibhatla I., Kumar S., Raghuwanshi S., Pallepati A., Gutti R.K. (2019). Long non-coding RNA: Classification, biogenesis and functions in blood cells. Mol. Immunol..

[28] Liu S., Liu X., Li J., Zhou H., Carr M.J., Zhang Z., Shi W. (2019). Long noncoding RNAs: Novel regulators of virus-host interactions. Rev. Med. Virol..

[29] Lin Y.H., Wu M.H., Yeh C.T., Lin K.H. (2018). Long Non-Coding RNAs as Mediators of Tumor Microenvironment and Liver Cancer Cell Communication. Int. J. Mol. Sci..

[30] Kung J.T., Colognori D., Lee J.T. (2013). Long noncoding RNAs: Past, present, and future. Genetics.

[31] Kolenda T., Guglas T., Ryś M., Bogaczyńska M., Teresiak A., Bliźniak R., Łasińska I., Mackiewicz J., Lamperska K. (2017). Biological role of long non-coding RNA in head and neck cancers. Rep. Pract. Oncol. Radiother..

[32] Kolenda T., Guglas K., Kopczyńska M., Teresiak A., Bliźniak R., Mackiewicz A., Lamperska K., Mackiewicz J. (2019). Oncogenic Role of ZFAS1 lncRNA in Head and Neck Squamous Cell Carcinomas. Cells.

[33] Kolenda T., Kopczyńska M., Guglas K., Teresiak A., Bliźniak R., Łasińska I., Mackiewicz J., Lamperska K. (2018). EGOT lncRNA in head and neck squamous cell carcinomas. Pol. J. Pathol..

[34] Jin J., Wu X., Yin J., Li M., Shen J., Li J., Zhao Y., Zhao Q., Wu J., Wen Q. (2019). Identification of Genetic Mutations in Cancer: Challenge and Opportunity in the New Era of Targeted Therapy. Front. Oncol..

[35] National Comprehensive Cancer Network Website. https://www.nccn.org.

[36] Malone E., Siu L.L. (2018). Precision Medicine in Head and Neck Cancer: Myth or Reality?. Clin. Med. Insights Oncol..

[37] Goossens N., Nakagawa S., Sun X., Hoshida Y. (2015). Cancer biomarker discovery and validation. Transl. Cancer Res..

[38] Lianidou E., Pantel K. (2019). Liquid biopsies. Genes Chromosomes Cancer.

[39] Sole C., Arnaiz E., Manterola L., Otaegui D., Lawrie C.H. (2019). The circulating transcriptome as a source of cancer liquid biopsy biomarkers. Semin. Cancer Biol..

[40] Eisenauer E.A., Therasse P., Bogaerts J., Schwartz L., Sargent D., Ford R., Dancey J., Arbuck S., Gwyther S., Mooney M. (2009). New response evaluation criteria in solid tumors: Revised RECIST guideline (version 1.1). Eur. J. Cancer Suppl..

[41] Lippi G. (2015). Systematic Assessment of the Hemolysis Index: Pros and Cons. Adv. Clin. Chem..

[42] Kolenda T., Ryś M., Guglas K., Teresiak A., Bliźniak R., Mackiewicz J., Lamperska K. (2019). Quantification of long non-coding RNAs using qRT-PCR: Comparison of different cDNA synthesis methods and RNA stability. Arch. Med. Sci..

[43] Thomas G.R., Shnayder Y., Willard H.F. (2010). Chapter 40—genomic evaluation of head and neck cancer A2—ginsburg, geoffrey S. Essentials of Genomic and Personalized Medicine.

[44] Kolenda T., Guglas K., Baranowski D., Sobocińska J., Kopczyńska M., Teresiak A., Bliźniak R., Lamperska K. (2020). cfRNAs as biomarkers in oncology—Still experimental or applied tool for personalized medicine already?. Rep. Pract. Oncol. Radiother..

[45] Yao Y., Chen X., Lu S., Zhou C., Xu G., Yan Z., Yang J., Yu T., Chen W., Qian Y. (2018). Circulating Long Noncoding RNAs as Biomarkers for Predicting Head and Neck Squamous Cell Carcinoma. Cell. Physiol. Biochem..

[46] Lu C.W., Zhou D.D., Xie T., Hao J.L., Pant O.P., Lu C.B., Liu X.F. (2018). HOXA11 antisense long noncoding RNA (HOXA11-AS): A promising lncRNA in human cancers. Cancer Med..

[47] Xue J.Y., Huang C., Wang W., Li H.B., Sun M., Xie M. (2018). *HOXA11-AS*: A novel regulator in human cancer proliferation and metastasis. Onco Targets Ther..

[48] Thin K.Z., Tu J.C., Raveendran S. (2019). Long non-coding SNHG1 in cancer. Clin. Chim. Acta.

[49] Lin S.X., Jiang H., Xiang G.Z., Zhang W.R., Weng Y.H., Qiu F.D., Wu J., Wang H.G. (2018). Up-regulation of long non-coding RNA SNHG1 contributes to proliferation and metastasis in laryngeal squamous cell carcinoma. Eur. Rev. Med. Pharmacol. Sci..

[50] Gao L., Cao H., Cheng X. (2018). A positive feedback regulation between long noncoding RNA SNHG1 and YAP1 modulates growth and metastasis in laryngeal squamous cell carcinoma. Am. J. Cancer Res..

[51] Wang L., Su K., Wu H., Li J., Song D. (2019). LncRNA SNHG3 regulates laryngeal carcinoma proliferation and migration by modulating the miR-384/WEE1 axis. Life Sci..

[52] Tang Y., Wu L., Zhao M., Zhao G., Mao S., Wang L., Liu S., Wang X. (2019). *LncRNA SNHG4* promotes the proliferation, migration, invasiveness, and epithelial-mesenchymal transition of lung cancer cells by regulating *miR-98-5p*. Biochem. Cell Biol..

[53] Liu D., Wang Y., Zhao Y., Gu X. (2020). LncRNA SNHG5 promotes nasopharyngeal carcinoma progression by regulating miR-1179/HMGB3 axis. BMC Cancer.

[54] Zhang S., Qiu D., Xie X., Shen Y. (2020). Clinicopathological and prognostic value of SNHG6 in cancers: A systematic review and a meta-analysis. BMC Cancer.

[55] Zhao Y., Wang J., Ma K. (2018). Knockdown of lncRNA SNHG6 Inhibites the Proliferation and Epithelial Mesenchymal Transition in Tongue Cancer Cells. Xi Bao Yu Fen Zi Mian Yi Xue Za Zhi.

[56] Guo T., Zambo K.D.A., Zamuner F.T., Ou T., Hopkins C., Kelley D.Z., Wulf H.A., Winkler E., Erbe R., Danilova L.V. (2020). Chromatin structure regulates cancer-specific alternative splicing events in primary HPV-related oropharyngeal squamous cell carcinoma. Epigenetics.

[57] Martinez B.V., Dhahbi J.M., Lopez Y.O.M., Lamperska K., Golusinski P., Luczewski L., Kolenda T., Atamna H., Spindler S.R., Golusinski W. (2015). Circulating small non-coding RNA signature in head and neck squamous cell carcinoma. Oncotarget.

[58] Kolenda T., Rutkowski P., Michalak M., Kozak K., Guglas K., Ryś M., Galus Ł., Woźniak S., Ługowska I., Gos A. (2019). Plasma lncRNA expression profile as a prognostic tool in BRAF-mutant metastatic melanoma patients treated with BRAF inhibitor. Oncotarget.

[59] Guglas K., Kolenda T., Stasiak M., Kopczyńska M., Teresiak A., Ibbs M., Bliźniak R., Lamperska K. (2020). YRNAs: New Insights and Potential Novel Approach in Head and Neck Squamous Cell Carcinoma. Cells.

[60] Guglas K., Kołodziejczak I., Kolenda T., Kopczyńska M., Teresiak A., Sobocińska J., Bliźniak R., Lamperska K. (2020). YRNAs and YRNA-Derived Fragments as New Players in Cancer Research and Their Potential Role in Diagnostics. Int. J. Mol. Sci..

[61] Drewell R.A., Arney K.L., Arima T., Barton S.C., Brenton J.D., Surani M.A. (2002). Novel conserved elements upstream of the H19 gene are transcribed and act as mesodermal enhancers. Development.

[62] Song C., Song C., Chen K., Zhang X. (2017). Inhibition of long non-coding RNA IGF2AS protects apoptosis and neuronal loss in anesthetic-damaged mouse neural stem cell derived neurons. Biomed. Pharmacother..

[63] Zhang X., Zhang X., Hu R., Hao L. (2019). Retraction: Prognostic implication and functional role of long noncoding RNA IGF2AS in human non-small cell lung cancer. J. Cell. Biochem..

[64] Cohen N., Fedewa S., Chen A.Y. (2018). Epidemiology and demographics of the head and neck cancer population. Oral Maxillofac. Surg. Clin. N. Am..

[65] Fayda M., Isin M., Tambas M. (2016). Do circulating long non-coding RNAs (lncRNAs) (LincRNA-p21, GAS 5, HOTAIR) predict the treatment response in patients with head and neck cancer treated with chemoradiotherapy?. Tumour Biol..

[66] Chen H., Xin Y., Zhou L. (2014). Cisplatin and paclitaxel target significant long noncoding RNAs in laryngeal squamous cell carcinoma. Med. Oncol..

[67] Ren S., Li G., Liu C. (2016). Next generation deep sequencing identified a novel lncRNA n375709 associated with paclitaxel resistance in nasopharyngeal carcinoma. Oncol. Rep..

[68] Li L., Gu M., You B. (2016). Long non-coding RNA ROR promotes proliferation, migration and chemoresistance of nasopharyngeal carcinoma. Cancer Sci..

[69] Tasheva E.S., Roufa D.S. (1995). Regulation of human RPS14 transcription by intronic antisense RNAs and ribosomal protein S14. Genes Dev..

[70] Tang Z., Wei G., Zhang L., Xu Z. (2019). Signature microRNAs and long noncoding RNAs in laryngeal cancer recurrence identified using a competing endogenous RNA network. Mol. Med. Rep..

[71] Liang K., Yang Y., Zha D., Yue B., Qiu J., Zhang C. (2018). Overexpression of lncRNA snaR is correlated with progression and predicts poor survival of laryngeal squamous cell carcinoma. J. Cell. Biochem..

