# Supplementary material for: Liquid lncRNA Biopsy for the Evaluation of Locally Advanced and Metastatic Squamous Cell Carcinomas of the Head and Neck
Source: J Pers Med. 2020 Sep 16;10(3):131. doi: 10.3390/jpm10030131 (PMC7564176; doi:10.3390/jpm10030131)
Supplement: Supplementary file 1 [file jpm-10-00131-s001.pdf]

SUPPLEMENTARY MATERIALS:

Suppl. Table 1. Baseline characteristic of patients included into the study. [TNM classification according to the 7th edition AJCC (because the 8th edition was effective since 2018), Other - salivary gland, CUP (carcinoma of unknown primary). In the induction group Tx means CUP; in the palliative group Tx means lack of primary site of tumor due to previous resection.]

**Parameter**	**Induction chemotherapy**	**Palliative chemotherapy**
	n (%)	n (%)
**All cases in study:**	17 (32%)	36 (68%)
**Localization of primary tumor:**		
Pharynx	9 (53%)	4 (11%)
Oral cavity	3 (18%)	13 (36%)
Larynx	4 (24%)	13 (36%)
Other	1 (5%)	6 (17%)
**T-stage:**		
T1	0	0
T2	3 (18%)	1 (3%)
T3	8 (48%)	9 (25%)
T4	4 (24%)	17 (47%)
Tx	2 (10%)	9 (25%)
**N-stage:**		
N0	0	8 (23%)
N1	0	4 (11%)
N2	12 (70%)	7 (19%)
N3	5 (30%)	17 (47%)
**Metastasis:**		
M0	17 (100%)	28 (77%)
M1	0	8 (33%)
Lungs	0	4 (50%)
Bones	0	2 (25%)
Liver	0	2 (25%)
**Stage:**		
III	3 (17%)	0

IVA	14 (83%)	0
IVC	0	36 (100%)
**Nicotinism:**	9 (52%)	16 (44%)
**Alcoholism:**	6 (35%)	16 (44%)
**Sex:**		
Female	11 (21%)	
Male	42 (79%)	
**Mean Age (years):**	60	
Female	61.5	
Male	55.1	

Suppl. Table 2. Treatment response as defined by RECIST 1.1; CR – complete response, PR – partial response, SD – stable disease, PD – progressive disease, DCR – disease control rate, RR – response rate.

**Response**	**Induction chemotherapy, n (%)**	**Palliative chemotherapy, n (%)**
CR	1 (6%)	0
PR	16 (94%)	4 (11%)
RR	17 (100%)	4 (11%)
SD	0 (0%)	3 (8%)
DCR	17 (100%)	7 (20%)
PD	0 (0%)	29 (80%)

Suppl. Tab. 3. Examined lncRNAs and reference genes ncProfiler qPCR Array Kit (System Biosciences).

Reference genes	18S rRNA. RNU43. GAPDH. LAMIN A/C. U6
	21A. 7SK. 7SL. Air. AK023948. Alpha 280. Alpha 250. ANRIL. anti-NOS2A. antiPeg11. BACE1AS. BC200. CAR Intergenic 10. DHFR upstream transcripts. Dio3os. DISC2. DLG2AS. E2F4 antisense. EgoA. EgoB. Emx2os. Evf1 and Evf2. GAS5-family.

Tested lncRNA	Gomafu. H19. H19 antisense. H19 upstream conserved 1&2. HAR1A. HAR1B. HOTAIR. HOTAIR1M. HOTTIP. Hoxa11as. HOXA3as. HOXA6as. HULC. IGF2AS. IPW. Jpx. Kcnq1ot1. KRASP1. L1PA16. p21. RoR. SFMBT2. VLDLR. LOC285194. LUST. Malat1. mascRNA. MEG3. MEG9. MER11C. ncR-uPAR. NDM29. NEAT1. Nespas. NRON. NTT. p53 mRNA. PCGEM1. PR antisense transcripts. PRINS. PSF inhibiting RNA. PTENP1. RNCR3. SAF. SCA8. snaR. SNHG1. SNHG3. SNHG4. SNHG5. SNHG6. Sox2ot. SRA. ST7OT. TEA ncRNAs. Tmevpg1. TncRNA. Tsix. TUG1. UCA1. UM9-5. WT1-AS. Xist. Y RNA-1. Zeb2NAT. Zfas1. Zfhx2as

Suppl. Tab. 4. Median expression of lncRNA in healthy volunteers and HNSCC patients with Confidence Interval (CI), and prognostic value of lncRNAs with sensitivity and specificity features to distinguish the examined groups; AUC – Area Under Curve.

lncRNA	lncRNA expression in healthy volunteers	lncRNA expression in HNSCC patients	P value	AUC	Sensitivity [%]	Specificity [%]
**21A**	1.6 [95%CI 1.04-3.9]	25.97 [95%CI 2.58-152.7]	0.0014	0.846	76.9	85.7
**Air**	0.004 [95% CI 0.002-0.0066]	0.33 [95% CI 0.09-1.16]	0.000014	0.863	61.5	100
**ANRIL**	0.000026 [95% CI 0.00001-0.00002]	0.0107 [95% CI 0.0011-472258002]	0.0018	1.000	100	100
**Alpha 280**	0.00980 [95% CI 0.0036-0.0180]	0.300 [95% CI 0.0277-1.195]	0.00066	0.874	88.5	85.7
**BACE1AS**	0	0.097 [95% CI 1.193-1.338	0.001	0.865	73.1	100
**Dio3os**	0.0950 [95% CI 0.0447-0.4314]	0.8931 [95% CI 0.2456-2.4296]	0.008	0.786	76.9	85.7
**E2F4 antisense**	0.0138 [95% CI 0.0056-0.0454]	0.5468 [95% CI 0.0404-1.3539]	0.0095	0.758	80.8	71.4
**Emx2os**	0.0346 [95% CI 0.011-0.1450]	0.1698 [95% CI 0.0420-1.12245]	0.034	0.703	88.5	57.1
**EvF1 and EVF2**	0.0257 [95% CI 0.0059-0.1905]	0.2411 [95% CI 0.0213-1.19508]	0.024	0.577	50	100
**GAS5**	0.5613 [95% CI 0.1641-0.8865]	9.9744 [95% CI 1.1632-67.8493]	0.00001	0.918	76.9	100
**H19**	0.10318 [95% CI 0.0682-0.2420]	1.0707 [95% CI 0.1651-5.4048]	0.0018	0.797	80.8	85.7
**HOXA6as**	0.3 [95% CI 0.2-0.5]	1.1138 [95% CI 0.4319-	0.0069	0.637	80.8	71.4

		5.1591]				
**Jpx**	0.0126 [95% CI 0.0048-0.055]	0.1237 [95% CI 0.0434-1.1466]	0.0031	0.769	96.2	57.1
**Kcnq1ot1**	0.0183 [95% CI 0.009-0.0529]	0.56199 [95% CI 0.0262-4.9670]	0.0062	0.714	69.2	85.7
**lincRNA-RoR**	0.0001 [95% CI 0.00002-0.000131]	2.1855 [95% CI 0.9112-8.8642]	0.00025	1.000	100	100
**mascRNA**	0.0615 [95% CI 0.0252-0.1934]	0.581 [95% CI 0.1209-1.1701]	0.0045	0.632	46.2	100
**MER11C**	0.0323 [95% CI 0.012-0.1071]	0.408 [95% CI 0.0732-1.0713]	0.0007	0.769	53.8	100
**ncR-uPAR**	0.0702 [95% CI 0.0048-0.1556]	1.0194 [95% CI 0.2202-6.7254]	0.0001	0.896	80.8	85.7
**NRON**	0.00002 [95% CI 0.000010-0.04038]	0.2801 [95% CI 0.0697-1.1106]	0.00008	0.885	100	71.4
**p53mRNA**	0.0104 [95% CI 0.00001-0.0403]	0.0894 [95% CI 0.0234-1.1691]	0.0068	0.901	73.1	100
**RNCR3**	0.0407 [95% CI 0.0056-0.0822]	0.2772 [95% CI 0.0659-1.1246]	0.00079	0.758	88.5	57.1
**SCA8**	0.0562 [95% CI 0.0298-0.1309]	0.9441 [95% CI 0.2225-1.4108]	0.0004	0.819	80.8	85.7
**SNHG1**	0.0171 [95% CI 0.0008-0.0299]	0.3861 [95% CI 0.0369-1.1563]	0.0017	0.934	76.9	100
**SNHG3**	0.0001 [95% CI 0.00005-0.0319]	0.4251 [95% CI 0.0897-1.0834]	0.00002	0.890	88.5	85.7
**SNHG4**	0.837 [95% CI 0.5046-1.66728]	3.4342 [95% CI 0.9395-26.5690]	0.017	0.659	38.5	100
**SNHG6**	0.039 [95% CI 0.0087-0.0818]	0.2788 [95 % CI 0.0517-1.1681]	0.002	0.885	73.1	100
**SRA**	0.0165 [95% CI 0.0151-0.0455]	0.1588 [95 % CI 0.0233-1.2028]	0.007	0.808	88.5	71.4
**ST7OT**	0.0850 [95% CI 0.0523-0.1835]	0.8573 [95% CI 0.1442-1.4057]	0.003	0.841	84.6	71.4
**TEA ncRNAs**	0.0314 [95% CI 0.012-0.1109]	0.1689 [95% CI 0.0272-1.3993]	0.033	0.808	61.5	100
**TncRNA**	0.7832 [95% 0.2712-1.2397]	1.3397 [95% CI 0.8122-5.3024]	0.01	0.643	34.6	100
**UCA1**	0.0013 [95% CI 0.0001-0.0130]	0.0601 [95% CI 0.0015-1.1489]	0.01	0.879	88.5	85.7
**WT1-AS**	0.02 [95% CI 0.007-0.0581]	0.1026 [95% CI 0.0149-1.1187]	0.049	0.593	57.7	71.4
**Y RNA-1**	3.3115 [95% CI 0.3171-327.6]	434 [95% CI 46 -1624]	0.003	0.797	80.8	71.4
**Zfas1**	0.00005 [95% CI 0.000006-0.4620]	0.992 [95% CI 0.1972-4.0048]	0.001	0.879	100	71.4

Suppl. Tab. 5. Median expression of lncRNA in volunteers and palliative treated HNSCC patients with Confidence Interval (CI), and prognostic value of lncRNAs with sensitivity and specificity features to distinguish the examined groups; AUC – Area Under Curve.

lncRNA	lncRNA expression in healthy volunteers	lncRNA expression in HNSCC palliative patients	P value	AUC	Sensitivity [%]	Specificity [%]
**21A**	1.6586 [95% CI 1.044-3.938]	47 [95% CI 9- 167]	0.00008	0.857	83.3	85.7
**Air**	0.0042 [95% CI 0.0025-0.0066]	0.1795 [95% CI 0.0181-1.0443]	0.00008	0.905	77.8	100
**Alpha 280**	0.0098 [95% CI 0.0036-0.018]	0.0788 [95% CI 0.0135-0.4929]	0.007	0.754	77.8	100
**ANRIL**	0.00002 [95% CI 0.000016-0.000027]	0.0107 [95% CI 0.0018-944516003]	0.002	1.000	100	100
**BACE1AS**	0	0.0573 [95% CI 0.0064-0.6750]	0.001	0.778	55.6	100
**Dios3os**	0.0950 [95% CI 0.0447-0.4314]	0.7031 [95% CI 0.2026-3.5973]	0.017	0.802	61.1	100
**E2F4 antisense**	0.0138 [95% CI 0.0056-0.0454]	0.2538 [95% CI 0.0209-1.3239]	0.029	0.802	88.9	71.4
**Emx2os**	0.0346 [95% CI 0.011-0.1450]	0.1405 [95% CI 0.0420-0.5884]	0.046	0.675	50.0	100
**GAS5**	0.5613 [95% CI 0.164-0.8865]	17 [95% CI 4- 97]	0.000007	0.905	83.3	100
**H19**	0.1031 [95% CI 0.0682-0.2420]	1.7411 [95% CI 0.1894-21]	0.002	0.698	61.1	100
**HAR1B**	2.6451 [95% CI 2.056-6.2184	13 [95% CI 2.99- 62]	0.03	0.817	72.2	85.7
**HOXA6as**	0.3 [95 % CI 0.2049-0.5414]	2.0505 [95% CI 0.2737-7.625]	0.016	0.635	44.4	100
**HULC**	0.108 [95% CI 0.038-0.2069]	0.3966 [95% CI 0.1134-1.5194]	0.02	0.603	83.3	42.9
**Jpx**	0.0126 [95% CI 0.00486-0.0550]	0.0708 [95% CI 0.0349-0.1916]	0.019	0.675	38.9	100
**Kcnq1ot1**	0.0183 [95% CI 0.009-0.0529]	0.1225 [95% CI 0.0183-40]	0.043	0.643	72.2	57.1
**KRASP 1**	0.0112 [95% CI 0.0035-0.0482]	0.1008 [95% CI 0.0172-0.3928]	0.023	0.667	38.9	100
**lincRNA-RoR**	0.0001 [95% CI 0.00002-0.00013]	2.7 [95% CI 0.826-7.314]	0.0003	1.000	100	100
**LUST**	1.083 [95% CI 0.7918-1.98]	17 [95% CI 2.4- 122]	0.005	0.841	66.7	100

**MALAT 1**	0.0126 [95% CI 0.0087-0.0297]	0.4031 [95% CI 0.0110-236129020]	0.043	0.881	88.9	85.7
**mascRNA**	0.0615 [95% CI 0.0252-0.1934]	0.3858 [95% CI 0.1275-1.2428]	0.0059	0.698	55.6	85.7
**MEG9**	0.0973 [95% CI 0.00001-0.474]	0.4705 [95% CI 0.187-1.2538]	0.032	0.810	94.4	71.4
**MER11C**	0.0323 [95% CI 0.0121-0.1071]	0.3441 [95% CI 0.0617-0.8295]	0.0026	0.754	55.6	100
**ncR-uPAR**	0.0702 [95% CI 0.0048-0.1556]	2 [95% CI 0.3919- 11]	0.00027	0.778	77.8	85.7
**NEAT1**	0.9351 [95% CI 0.6893-1.753	3 [95% CI 0.9866- 20]	0.012	0.857	72.2	85.7
**NRON**	0.00002 [95% CI 0.00001-0.0403]	0.2799 [95% CI 0.0688-0.6151]	0.0002	0.841	100	57.1
**P53mRNA**	0.0104 [95% CI 0.00001-0.0403]	0.0524 [95% CI 0.0175-0.1426]	0.023	0.817	83.3	71.4
**PSF inhibiting RNA**	0.00005 [95% CI 0.00003-0.00114]	0.0069 [95% CI 0.0009-0.1494]	0.034	0.960	83.3	100
**PTENP1**	0.0002 [95% CI 0.00002-0.0201]	0.0142 [95% CI 0.0007-0.3258]	0.046	0.714	83.3	71.4
**RNCR3**	0.0407 [95% CI 0.0056-0.0822]	0.2609 [95% CI 0.0773-1.010]	0.0016	0.706	77.8	71.4
**SCA8**	0.0562 [95% CI 0.0298-0.1309]	0.5062 [95% CI 0.1981-2.1583]	0.002	0.698	72.2	71.4
**SNHG1**	0.0171 [95% CI 0.0008-0.0299]	0.3728 [95% CI 0.0823-1.1076]	0.0005	0.921	77.8	100
**SNHG3**	0.0001 [95% CI 0.000059-0.0319]	0.2157 [95% CI 0.0457-1.0730]	0.0003	0.881	66.7	100
**SNHG4**	0.837 [95% CI 0.5046-1.6672]	8 [95% CI 1 – 49]	0.0044	0.714	88.9	57.1
**SNHG6**	0.039 [95% CI 0.0087-0.0818]	0.2549 [95% CI 0.0524-0.7123]	0.006	0.873	72.2	100
**SRA**	0.0165 [95% CI 0.0151-0.0455]	0.0877 [95% CI 0.0219-0.612]	0.029	0.770	83.3	71.4
**ST7OT**	0.085 [95% CI 0.0523-0.1835]	0.6983 [95% CI 0.1854-3.1712]	0.0027	0.944	88.9	100
**TncRNA**	0.7832 [95% CI 0.2712-1.2397]	2.9554 [95% CI 0.8122-9]	0.0038	0.778	61.1	100
**Tsix**	2.6515 [95% CI 2.37-3.832]	11.77 [95% CI 3.132-48.4]	0.37	0.857	94.4	71.4
**UCA1**	0.0013 [95% CI 0.0001-0.0130]	0.0259 [95% CI 0.0013-0.1689]	0.039	0.881	83.3	85.7
**Y RNA-1**	3.311 [95% CI 0.3171-327]	561 [95% CI 261 – 2509]	0.0007	0.865	88.9	85.7

**Zfas1**	0.00005 [95% CI 0.000006-0.4620]	1.0281 [95% CI 0.08963- 5.3517]	0.002	0.778	100	57.1

Suppl. Tab. 6. Median expression of lncRNA in volunteers and locally advanced HNSCC patients with Confidence Interval (CI), and prognostic value of lncRNAs with sensitivity and specificity features to distinguish the examined groups; AUC – Area Under Curve.

**lncRNA**	**lncRNA expression in healthy volunteers**	**lncRNA expression in HNSCC locally advanced patients**	**P value**	**AUC**	**Sensitivity [%]**	**Specificity [%]**
**7SK**	0.1174 [95% CI 0.0741-0.3385]	1.096 [95% CI (1.028-1.1218]	0.029	1.000	100	100
**Air**	0.0042 [95% CI 0.0025-0.0066]	1.1404 [95% CI 0.1956-1.2466]	0.00008	0.768	75.0	71.4
**Alpha 280**	0.0098 [95% CI 0.0036-0.0180]	1.1354 [95% CI 0.3003-1.2678]	0.0001	0.839	62.5	100
**antiPeg11**	0.0830 [95% CI 0.0273-0.1682]	1.0409 [95% CI 0.11-1.1724]	0.017	0.714	62.5	71.4
**BACE1AS**	0	1.066 [95% CI 0.0215-1.2067]	0.001	0.875	75.0	100
**BC200**	0.3186 [95% CI 0.2246-0.4553]	1.03996 [95% CI 0.9838-1.0859]	0.01	0.714	50.0	100
**DHFR upstream transcripts**	0.0792 [95% CI 0.06168-0.348]	1.0138 [95% CI 0.1568-1.0834]	0.041	0.839	100	71.4
**Dio3os**	0.09508 [95% CI 0.0447-0.4314]	0.9387 [95% CI 0.5224-1]	0.01	0.696	75.0	71.4
**DISC2**	0.0317 [95% CI 0.0031-0.081]	1.1733 [95% CI 0.286-1.2092]	0.026	0.893	75.0	100
**E2F4 antisense**	0.0138 [95% CI 0.0056-0.0454]	1.1089 [95% CI 0.5468-1.3538]	0.0047	0.786	75.0	100
**Evf1 and EVF2**	0.0257 [95% CI 0.0059-0.1905]	1.03 [95% CI 0.063-1.187]	0.009	0.554	10.0	100
**GAS5**	0.5613 [95% CI 0.1641-0.8865]	1.1027 [95% CI 0.9088-12]	0.001	0.732	100	71.4
**H19**	0.1031 [95% CI 0.0682-0.2420]	0.9385 [95% CI 0.1202-1.0641]	0.018	0.714	62.5	100
**H19 upstream conserved 1 i 2**	3 [95% CI 1.870-12.1889]	0.9889 [95% CI 0.9487-1.9251]	0.007	0.821	75	100
**Hoxa11as**	2.8828 [95% CI 1.34356-4.4485]	0.8923 [95% CI 0.6080-0.94623]	0.012	0.911	87.5	100
**HOXA3as**	0.0051 [95% CI 0.0006-0.0181]	1.163 [95% CI 0.0477-1.287]	0.027	0.839	62.5	100
**HOXA6as**	0.3 [95% CI 0.2049-0.5414]	1.084 [95% CI 0.97683-1.205]	0.01	0.768	62.5	100

**IPW**	0.0325 [95% CI 0.0068-0.0888]	1.1788 [955 CI 1.0828-1.264]	0.013	0.946	87.5	100
**Jpx**	0.0126 [95% CI 0.0048-0.055]	1.1455 [95% CI 1.0504-1.1785]	0.0006	0.911	87.5	100
**Kcnq1ot1**	0.0183 [95% CI 0.009-0.0529]	1.0286 [95% CI 0.14935-1.1492]	0.0009	0.643	50.0	100
**KRASP1**	0.0112 [95% CI 0.0035-0.0482]	1.0658 [95% CI 0.07807-1.1151]	0.008	0.750	62.5	100
**Linc-RoR**	0.0001 [95% CI 0.00002-0.00013]	1.0087 [95% CI 0.9451-8.9]	0.0009	1.00	100	100
**lincRNA-SFMBT2**	0.0066 [95% CI 0.0009-0.0571]	0.8462 [95% CI 0.02904-1.2009]	0.035	0.714	62.5	100
**LOC285194**	0.046 [95% CI 0.0111-0.1132]	1.0529 [95% CI 0.68-1.1719]	0.032	0.911	75	100
**Malat1**	0.0126 [95% CI 0.0087-0.0297]	1.2149 [95% CI 0.4363-1.235]	0.026	1.000	100	100
**mascRNA**	0.0615 [95% CI 0.0252-0.1934]	1.0818 [95% CI 0.1047-1.1653]	0.026	0.661	50.0	100
**MEG9**	0.0973 [95% CI 0.00001-0.474]	1.043 [95% CI 0.275-1.104]	0.014	0.875	100	71.4
**MER11C**	0.0323 [95% CI 0.0121-0.1071]	1.0546 [95% CI 0.1666-1.0855]	0.0016	0.696	50.0	100
**ncR-uPAR**	0.0702 [95% CI 0.0048-0.1556]	0.9877 [95% CI 0.1929-1.0194]	0.002	0.607	37.5	100
**NRON**	0.00002 [95% CI 0.00001-0.0403]	1.0202 [95% CI 0.0875-1.1396]	0.00009	0.911	87.5	87.5
**P53 mRNA**	0.0104 [95% CI 0.0026-0.0336]	1.16769 [95% CI 0.3666-1.2498]	0.0045	0.929	75.0	100
**PCGEM1**	0.0332 [95% CI 0.0079-0.0835]	1.1589 [95% CI 1.1297-4367666984761]	0.014	1.000	100	100
**PSF inhibiting RNA**	0.00005 [95% CI 0.00003-0.00114]	1.3466 [95% CI 1.2689-2183833492381]	0.019	1.000	100	100
**PTENP1**	0.0002 [95% CI 0.00002-0.0201]	1.066 [95% CI 0.7310-1.2724]	0.01	0.946	75.0	100
**RNCR3**	0.0407 [95% CI 0.0056-0.0822]	0.9958 [95% CI 0.0676-1.1167[	0.0044	0.500	37.5	100
**SCA8**	0.0562 [95% CI 0.0298-0.1309]	1.0567 [95% CI 0.9613-1.1102]	0.0005	0.821	62.5	100
**SNHG3**	0.0001 [95% CI 0.00005-0.0319]	1.0213 [95% CI 0.771-1.0776]	0.00001	1.000	100	100
**SNHG5**	0.0367 [95% CI 0.012-0.0979]	1.1734 [95% CI 0.2116-1.2404]	0.005	0.821	75.0	100
**SNHG6**	0.039 [95% CI 0.0087-0.0818]	1.0945 [95% CI 0.11489-1.1747]	0.0042	0.804	87.5	71.4

**Sox2ot**	0.0050 [95% CI 0.0010-0.0455]	1.111 [95% CI 0.6243-1.2679]	0.023	0.929	87.5	100
**SRA**	0.0165 [95% CI 0.0151-0.0455]	1.13911 [95% CI 0.0893-1.24080]	0.0021	0.875	62.5	100
**ST7OT**	0.0850 [95% CI 0.0523-0.1835]	1.0959 [95% CI 0.12133-1.1504]	0.032	0.679	37.5	100
**TEA ncRNAs**	0.0314 [95% CI 0.012-0.1109]	1.2614 [95% CI 1.0758-1.2684]	0.0017	0.946	87.5	100
**UCA1**	0.0013 [95% CI 0.00018-0.01309]	1.1215 [95% CI 0.091-1.3391]	0.012	0.964	87.5	100
**WT1-AS**	0.02 [95% CI 0.007-0.0581]	1.1187 95% CI 0.4265-1.1834]	0.009	0.893	87.5	100
**Zfas1**	0.00005 [95% CI 0.000006-0.4620]	0.9772 [95% CI (0.4416-1.056]	0.007	0.839	100	71.4
**Zfhx2as**	0.2085 [95% CI 0.1262-0.4536]	1.0721 [95% CI 0.9180-2.1509]	0.007	0.983	100	85.7

Suppl. Tab. 7. lncRNA expression according to the primary site of tumor. Only statistically significant ($p < 0.05$) lncRNA due to localization of tumor are presented.

**Localization**	**lncRNA**	**P value**
pharynx *vs* oral cavity	HAR1B	0.0239
	Jpx	0.0464
	LUST	0.0043
	NEAT1	0.0340
	TncRNA	0.0414
pharynx *vs* larynx	Air	0.0414
pharynx *vs* salivary gland	21A	0.0046
	7SL	0.0046
	Alpha 280	0.0484
	DHFR upstream transcripts	0.0126
	Dio3os	0.0077
	EgoB	0.0055
	EgoA	0.0096
	GAS5	0.0013
	H19 upstream conserved 1and 2	0.0215
	HAR1B	0.0043
	HOTAIR	0.0201
	HOXA6as	0.0019
	LUST	0.0030
	NEAT1	0.0074
	Nespas	0.0323
	SCA8	0.0350
	SNHG4	0.0316
	TUG1	0.0070

	TncRNA	0.0006
	Y RNA-1	0.0089
	Zeb2NAT	0.0470
	Zfas1	0.0383
	anti-NOS2A	0.0015
	lincRNA-VLDLR	0.0215
	mascRNA	0.0281
oral cavity *vs* salivary gland	Alpha 280	0.0391
	DHFR upstream transcripts	0.0344
	Dio3os	0.0344
	GAS5	0.0034
	HAR1B	0.0415
	HOXA6as	0.0344
	LUST	0.0216
	NEAT1	0.0471
	SAF	0.0353
	TUG1	0.0176
	TncRNA	0.0029
	Zeb2NAT	0.0484
	Zfhx2as	0.0471
	anti-NOS2A	0.0265
	mascRNA	0.0228
larynx *vs* salivary gland	21A	0.0100
	Air	0.0202
	Alpha 280	0.0106
	DHFR upstream transcripts	0.0092
	Dio3os	0.0415
	E2F4 antisense	0.0393
	EGO B	0.0353
	GAS5	0.0008
	H19 upstream conserved 1and 2	0.0298
	HAR1B	0.0489
	HOTAIR	0.0388
	HOXA6as	0.0110
	LUST	0.0391
	SAF	0.0353
	TUG1	0.0076
	TncRNA	0.0042
	Y RNA-1	0.0262
	Zfas1	0.0298
	Zfhx2as	0.0070
	anti-NOS2A	0.0211
	lincRNA-VLDLR	0.0187
	mascRNA	0.0100
